# Lack of diuresis four hours prior to admission is associated with increased mortality in acutely admitted medical patients

**DOI:** 10.1186/1757-7241-23-S1-A35

**Published:** 2015-07-16

**Authors:** Mikkel Brabrand, Daniel P Henriksen

**Affiliations:** 1 Emergency Department, Sydvestjysk Sygehus, Esbjerg, Denmark; 2 Emergency Department, OUH Odense University Hospital, Odense, Denmark; 3 Institute of Regional Health Research, University of Southern Denmark, Odense, Denmark; 4 Department of Clinical Chemistry and Pharmacology, Odense University Hospital, Odense, Denmark

## Background

When patients become hypotensive, the kidneys and brain are the first organs to be affected. A key symptom of hypo-perfusion of the kidneys is reduced diuresis. Patients will experience this as less frequent visits to the toilet. The aim of the study was to assess if there was an association between reduced frequency of toilet visits and short-term mortality.

## Methods

We included all consecutively admitted medical patients to the medical admission unit at Sydvestjysk Sygehus Esbjerg from October 2008 through February 2009. Upon arrival, a nurse registered their demographic details as well as vital signs and whether they had had any diuresis within the last four hours. After all patients had either died or been discharged, we extracted survival status from the Danish Civil Register. We calculated 1- and 7-day survival and using logistic regression, assessed the crude association between diuresis within the last four hours before admission and mortality. We also performed analyses to assess the association adjusting for age, systolic blood pressure and Charlson Comorbidity Index (CCI), all as continuous variables.

## Results

We included 3,046 patients. 1,460 (48%) were female, and the median age was 66 (range 15-107) years. Information on recent diuresis was available for 1,859 (61%) patients. Of the 1,859 patients with information on diuresis, 283 (15%) experienced no diuresis within four hours up to arrival. The 1-day mortality was 0.8 (95% Confidence interval (CI): 0.5-1.3)%. Patients without diuresis within the last four hours had a crude odds ratio (OR) of 23.2 (95% CI: 6.5-82.8). Adjusting for age, systolic blood pressure, and CCI resulted in an OR of 18.0 (95% CI: 5.0-65.1). We found a 7-day mortality of 2.3% (95% CI: 1.7-3.1). Crude OR for 7-day mortality was 4.2 (95% CI: 2.3-7.8) and adjusted OR was 3.3 (95% CI: 1.7-6.2).

## Conclusions

Among patients with a recording of diuresis, those without any diuresis within four hours before admission, have a significantly increase 1- and 7-day mortality compared to patients who have had diuresis. Our results are limited by small numbers of patients meeting the outcome.

